# ADAM10-Interacting Tetraspanins Tspan5 and Tspan17 Regulate VE-Cadherin Expression and Promote T Lymphocyte Transmigration

**DOI:** 10.4049/jimmunol.1600713

**Published:** 2017-06-09

**Authors:** Jasmeet S. Reyat, Myriam Chimen, Peter J. Noy, Justyna Szyroka, G. Ed Rainger, Michael G. Tomlinson

**Affiliations:** * School of Biosciences, College of Life and Environmental Sciences, University of Birmingham, Birmingham B15 2TT, United Kingdom; and; † Institute of Cardiovascular Sciences, College of Medical and Dental Sciences, University of Birmingham, Birmingham B15 2TT, United Kingdom

## Abstract

The recruitment of blood leukocytes across the endothelium to sites of tissue infection is central to inflammation, but also promotes chronic inflammatory diseases. A disintegrin and metalloproteinase 10 (ADAM10) is a ubiquitous transmembrane molecular scissor that is implicated in leukocyte transmigration by proteolytically cleaving its endothelial substrates. These include VE-cadherin, a homotypic adhesion molecule that regulates endothelial barrier function, and transmembrane chemokines CX3CL1 and CXCL16, which have receptors on leukocytes. However, a definitive role for endothelial ADAM10 in transmigration of freshly isolated primary leukocytes under flow has not been demonstrated, and the relative importance of distinct ADAM10 substrates is unknown. Emerging evidence suggests that ADAM10 can be regarded as six different molecular scissors with different substrate specificities, depending on which of six TspanC8 tetraspanins it is associated with, but TspanC8s remain unstudied in leukocyte transmigration. In the current study, ADAM10 knockdown on primary HUVECs was found to impair transmigration of freshly isolated human peripheral blood T lymphocytes, but not neutrophils or B lymphocytes, in an in vitro flow assay. This impairment was due to delayed transmigration rather than a complete block, and was overcome in the presence of neutrophils. Transmigration of purified lymphocytes was dependent on ADAM10 regulation of VE-cadherin, but not CX3CL1 and CXCL16. Tspan5 and Tspan17, the two most closely related TspanC8s by sequence, were the only TspanC8s that regulated VE-cadherin expression and were required for lymphocyte transmigration. Therefore endothelial Tspan5- and Tspan17-ADAM10 complexes may regulate inflammation by maintaining normal VE-cadherin expression and promoting T lymphocyte transmigration.

## Introduction

Leukocyte recruitment during inflammation is essential for fighting infection and repairing tissue damage. Ordinarily this process is under tight control, because loss of regulation can result in the prolonged and inappropriate patterns of leukocyte trafficking, which result in chronic inflammatory diseases such as atherosclerosis. During inflammation, leukocytes are captured from the bloodstream by adhesion molecules on endothelial cells of postcapillary venules (1–4). The leukocytes then undergo a process of rolling, arrest, crawling, and transendothelial migration. The early events of this process are relatively well-characterized, but transendothelial migration is less well understood (1–4).

A disintegrin and metalloproteinase 10 (ADAM10) is a ubiquitous transmembrane molecular scissor, or sheddase, which cleaves the extracellular regions from over 40 different transmembrane substrates. Endothelial ADAM10 has the potential to regulate leukocyte transmigration as a sheddase for the adherens junction protein VE-cadherin (5, 6) and the transmembrane chemokines CX3CL1 and CXCL16 (7–9). Indeed, three in vitro studies report a role for endothelial ADAM10 in leukocyte transmigration. In the only one of these to use relevant populations of freshly isolated blood leukocytes, knockdown of ADAM10 expression on primary human lung microvascular endothelial cells was found to impair the transmigration of human neutrophils toward CXCL8/IL-8 (10). In the other two studies, pharmacological inhibition of ADAM10 activity, or knockdown of expression, on primary HUVECs was found to impair transmigration of cultured human T cells preactivated with the mitogen PHA (6), and to impair transmigration of a mouse pre-B cell line transfected with CX3CR1, the CX3CL1 receptor (11). However, the interpretation of experimental outcomes is complicated when differentiated cells and cell lines are used, as the patterning of adhesion and chemokine receptors will deviate from those found on cells circulating in the blood. The only study to have conducted assays with freshly isolated blood leukocytes under physiological flow conditions reports no significant role of endothelial ADAM10 on the transmigration of primary human monocytes (12). Thus, despite its potential to regulate leukocyte transmigration, endothelial ADAM10 is not generally included in current models of leukocyte transmigration (1–4).

We and others have recently discovered that ADAM10 is regulated by tetraspanins (13–15), which are a superfamily of 33 transmembrane proteins in mammals that regulate the intracellular trafficking and membrane localization of the so-called partner proteins with which they associate (16, 17). The ADAM10-interacting tetraspanins are the recently identified and relatively poorly characterized TspanC8 subgroup, which are related by sequence and comprise Tspan5, 10, 14, 15, 17, and 33 (13, 14). Interaction with a TspanC8 is required for ADAM10 exit from the endoplasmic reticulum, and for enzymatic maturation and trafficking to the cell surface (13–15). Moreover, emerging evidence indicates that different TspanC8s traffic ADAM10 to distinct subcellular localizations (13, 18) and can differentially affect cleavage of the ADAM10 substrates Notch (13, 18, 19), N-cadherin (13, 15, 20), GPVI (20), amyloid precursor protein (15, 18), and CD44 (18). Therefore, ADAM10 can be regarded as six different molecular scissors, depending on its associated TspanC8 (21). A potential role for specific TspanC8-ADAM10 complexes in leukocyte transmigration has not been investigated.

The aims of the current study were to determine whether endothelial ADAM10 promotes transmigration of freshly isolated primary human lymphocytes and neutrophils under physiological flow conditions, to identify the key ADAM10 substrate(s) involved, and to investigate whether specific TspanC8s regulate this process.

## Materials and Methods

### Cells

HUVECs were obtained from umbilical cords with consent from the Birmingham Women’s Health Care NHS Trust, which was approved by the University of Birmingham Ethics Committee. HUVECs were isolated using the collagenase digestion method (22) and cultured in M199 media supplemented with 20% FBS, 10 ng/ml epidermal growth factor (Sigma), 35 μg/ml gentamicin, 1 μg/ml hydrocortisone, and 2.5 μg/ml amphotericin B. Human PBLs and neutrophils were obtained from venous blood of healthy individuals, which was collected under ethical approval into EDTA tubes. PBMCs were isolated by centrifugation on Histopaque 1077 (Sigma), before being panned on culture plastic to remove adherent monocytes, and yield a population of PBLs (23). Neutrophils were isolated by centrifugation of blood on Histopaque 1119 (Sigma). Isolated cells were washed, counted, and adjusted to a final concentration of 1 × 10^6^ per ml in M199 media supplemented with 0.15% BSA for static adhesion assays, or in PBS with 0.15% BSA for flow adhesion assays. For experiments in which equal numbers of PBLs and neutrophils were mixed, the cells were labeled with 25 μM CellTracker Orange (Life Technologies) and 5 μg/ml calcein-AM (Cambridge Bioscience), respectively. The human embryonic kidney-293 cells expressing the large T-antigen of SV40 (HEK-293T) cell line was cultured in DMEM medium containing 10% FBS (Life Technologies), 4 mM l-glutamine, 100 U/ml penicillin, and 100 μg/ml streptomycin.

### Leukocyte transmigration under static conditions

PBL transmigration was assessed using a 12-well format as previously described (24). First passage HUVECs were seeded on to 12-well tissue culture plates at a density to yield a confluent monolayer within 24 h. The HUVECs were stimulated with 100 U/ml TNF-α (R&D Systems) and 10 ng/ml IFN-γ (PreproTech) for 24 h prior to carrying out adhesion assays with lymphocytes. HUVECs were washed with M199 0.15% BSA to remove residual cytokines and purified PBLs were added. The PBLs were allowed to settle, adhere, and transmigrate through the HUVEC monolayer at 37°C in a CO_2_ incubator for 7 min, a time point previously shown to yield a sufficient proportion of migrated PBLs (25). Nonadherent cells were removed from the HUVECs by gently washing with M199 0.15% BSA, and video recordings of five fields of view of the endothelial monolayer were made using phase-contrast video microscopy. The video recordings were analyzed using Image-Pro Plus software (DataCell). Each PBL was classified as either phase bright and adherent to the surface of the HUVEC monolayer, or phase dark with altered morphology and migrating below the HUVEC monolayer. The percentage of adherent PBL that had transmigrated was calculated.

### Leukocyte transmigration under flow

First passage HUVECs were seeded onto ibidi chamber slides (ibidi) at a density designed to yield a confluent monolayer 24 h later, and stimulated as described for the static assay. The ibidi chamber slides were attached to a perfusion system mounted on the stage of a phase-contrast video microscope enclosed in a Perspex chamber at 37°C, as described (26). At one end, the ibidi slide was connected to a Harvard withdrawal syringe pump that delivered the flow at a rate equivalent to a wall shear stress of 0.05 Pa. At the other end, the ibidi slide was connected to an electronic switching valve (Lee Products), which selected the flow from two reservoirs containing leukocytes (PBLs or neutrophils) in PBS 0.15% BSA or cell-free PBS 0.15% BSA. A 4 min bolus of leukocytes was perfused over the HUVECs followed by cell-free PBS 0.15% BSA wash buffer. Video recordings were made of a series of microscope fields along the center-line of the flow channel after 9 min of washout. Video recordings were analyzed as for static assays, except that leukocytes adherent to the HUVEC monolayer were classified as rolling (phase bright spherical cells moving over the surface), firmly adherent (phase bright cells with distorted shape and migrating slowly on the surface), or phase dark transmigrated cells. The total number leukocytes exhibiting the various behaviors was calculated and expressed as a percentage.

### Flow cytometry for transmembrane chemokines and their receptors

PBLs and monocytes were stained with anti-CD3 peridinin-chlorophyll proteins–Cy5.5, anti-CD4 allophycocyanin-Cy7, anti-CD8 Pacific Blue, anti-CD14 allophycocyanin, and anti-CD19 PE-Cy7 (BD Biosciences), and anti-CX3CR1 FITC and anti-CXCR6 FITC (R&D Systems). HUVECs were stained with anti-CX3CL1 FITC and anti-CXCL16 allophycocyanin (R&D Systems). As a positive control for these transmembrane chemokines, HEK-293T cells were transfected with expression constructs for human CX3CL1 (8) or CXCL16 (7), using polyethylenimine (Sigma) as described (27). HUVECs were also stained with anti-E-selectin PE, anti-ICAM-1 allophycocyanin and anti-VCAM-1 FITC (BD Biosciences). Cells were analyzed using a Cyan ADP Flow Cytometer (Beckman Coulter) and FlowJo software (Ashland). Live cells were gated based on forward and side scatter parameters.

### Small interfering RNA knockdown in HUVECs

Silencer Select small interfering RNA (siRNA) duplexes (Life Technologies) were transfected into HUVECs using Lipofectamine RNAiMAX (Life Technologies) as described (14), and functional assays were performed 48 h post transfection. ADAM10 and VE-cadherin knockdowns were assessed by flow cytometry using anti-ADAM10 FITC (R&D Systems) and anti-VE-cadherin allophycocyanin (BD Biosciences). TspanC8s knockdowns were assessed by TaqMan (Applied Biosystems) quantitative PCR using RNA isolated with the RNeasy Mini Kit (Qiagen), from which cDNA was made using the High Capacity cDNA Reverse Transcription Kit (Life Technologies), as described (14). TspanC8 data were normalized to quantitative PCR data for GAPDH.

### Statistical analyses

All percentage data were normalized by arcsine transformation before statistical testing by Student *t* test for two samples, or by ANOVA, followed by Bonferroni or Dunnett multiple comparison tests for more than two samples.

## Results

### Endothelial ADAM10 promotes PBL transmigration

**FIGURE 1.**
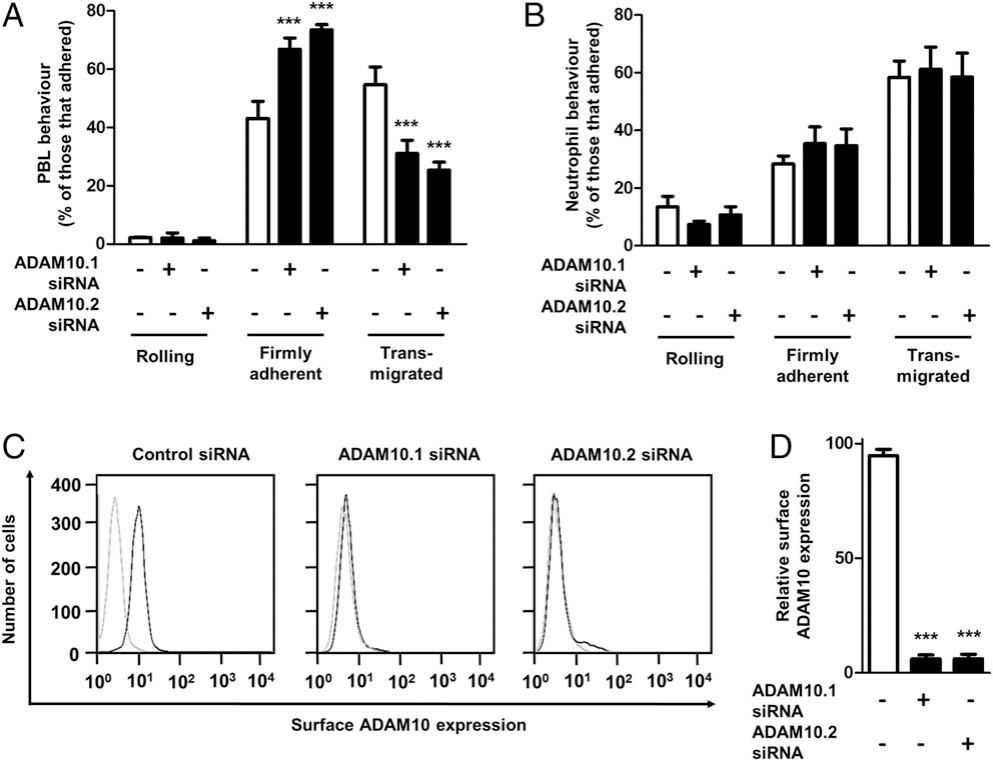
Knockdown of endothelial ADAM10 decreases the transmigration of lymphocytes under in vitro flow conditions. (**A**) HUVECs were transfected with 10 nM negative control (white bars) or one of two ADAM10 siRNA duplexes (black bars). HUVECs were replated into six-well ibidi slides 4 h post transfection, and stimulated with 100 U/ml TNF-α and 10 ng/ml IFN-γ 24 h post transfection. After a further 24 h, freshly isolated human PBLs were perfused across HUVEC monolayers at 0.05 Pa for 4 min, followed by cell-free PBS 0.15% BSA for 9 min. Video recordings of five different fields of view of the HUVEC monolayer were then made using time-lapse phase-contrast video microscopy. Cells were classified as rolling, firmly adherent, or transmigrated. Error bars represent the SEM from five experiments. Data were normalized by arcsine transformation and statistically analyzed by a two-way ANOVA and Bonferroni post hoc comparison test (***p < 0.001 compared with control siRNA-transfected cells). (**B**) The experiments were carried out as explained in (A), except that HUVECs were prestimulated with 100 U/ml TNF-α for 4 h before perfusion of human neutrophils. Error bars represent the SEM from seven experiments. (**C**) ADAM10 knockdown was confirmed by flow cytometry. The black line represents ADAM10 staining and the gray line isotype control staining from a representative experiment. (**D**) ADAM10 knockdown was quantitated and error bars represent the SEM from the 12 total experiments in (A) and (B). Data were normalized by arcsine transformation and analyzed by *t* test. ***p < 0.001 compared with control siRNA-transfected cells.

Previous studies have shown that endothelial ADAM10 is required for efficient transmigration of human neutrophils toward CXCL8 (10), cultured human T cells preactivated with the mitogen PHA (6), and a CX3CR1-transfected pre-B cell line (11) under static conditions. Under flow conditions, ADAM10 is not required for primary human monocyte transmigration (12). To test whether endothelial ADAM10 regulates primary human PBL or neutrophil transmigration under flow, an established in vitro assay using physiological flow conditions was employed (26), following siRNA knockdown of ADAM10 on HUVECs. ADAM10 knockdown reduced PBL transmigration by ∼50% with two different siRNA duplexes, with a consequent increase in the percentage of firmly adherent cells that had failed to transmigrate (Fig. 1A). In contrast, endothelial ADAM10 knockdown did not affect neutrophil transmigration (Fig. 1B). ADAM10 knockdown efficiency was confirmed by flow cytometry (Fig. 1C) and found to be ∼90% upon quantitation (Fig. 1D). These data show that endothelial ADAM10 is required for normal PBL, but not neutrophil, transmigration under physiological flow conditions in our in vitro model system driven by endothelial cell responses to inflammatory cytokines.

**FIGURE 2.**
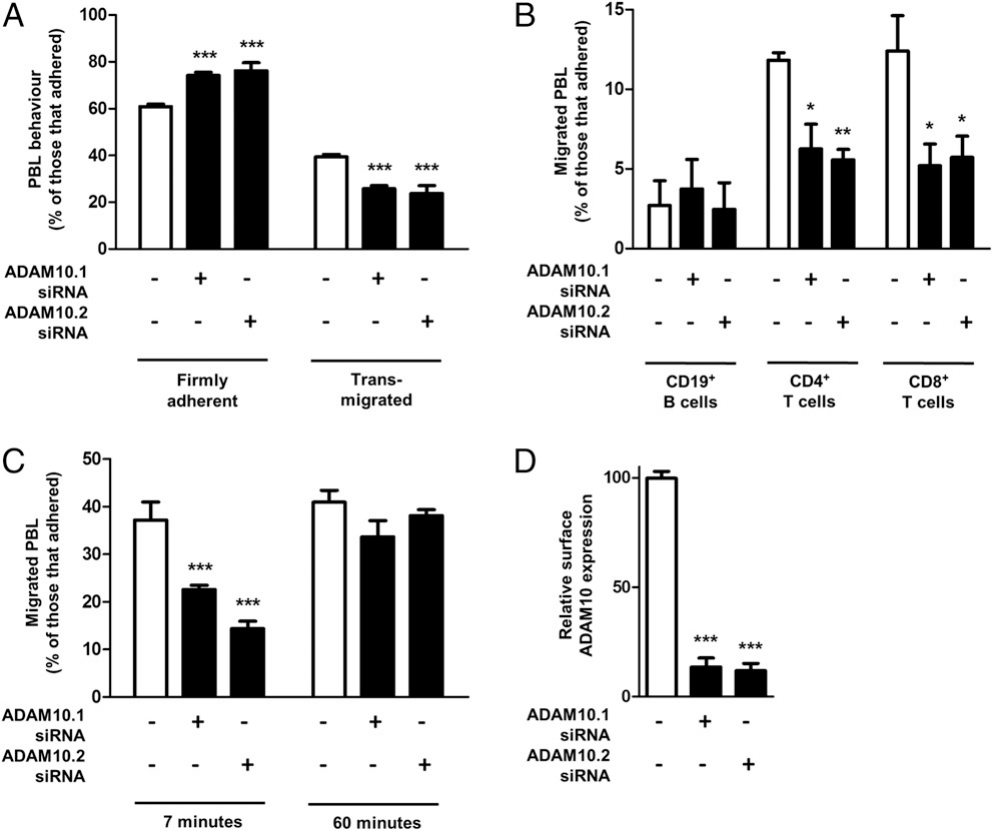
Knockdown of endothelial ADAM10 decreases the transmigration of lymphocytes under in vitro static conditions. (**A**) HUVECs were siRNA transfected and stimulated as described in Fig. 1A, but were cultured in 12-well plates. Freshly isolated human PBLs were allowed to adhere to HUVEC monolayers for 7 min at 37°C. Phase-contrast images of five different fields of view of the HUVEC monolayer were taken. Cells were classified as firmly adherent or transmigrated. Error bars represent the SEM from five experiments and statistical analyses were performed as described for Fig. 1A (***p < 0.001). (**B**) The experiment was performed as described in (A), except that transmigrated PBLs were harvested following initial removal of surface-adherent PBLs using 0.02% EDTA in PBS. Expression of major PBL subpopulations (CD19^+^ B cells, CD4^+^, and CD8^+^ T cells) was assessed by flow cytometry. Error bars represent the SEM from three experiments. *p < 0.05, **p < 0.01 compared with control siRNA-transfected cells. (**C**) The experiments were carried out as explained in (A), except that human PBLs were allowed to adhere to HUVEC monolayers for 7 or 60 min at 37°C. Error bars represent the SEM from three experiments. ***p < 0.001. (**D**) ADAM10 knockdown was quantitated and statistical analyses were performed as described in Fig. 1D. Error bars represent the SEM from five experiments. ***p < 0.001.

**FIGURE 3.**
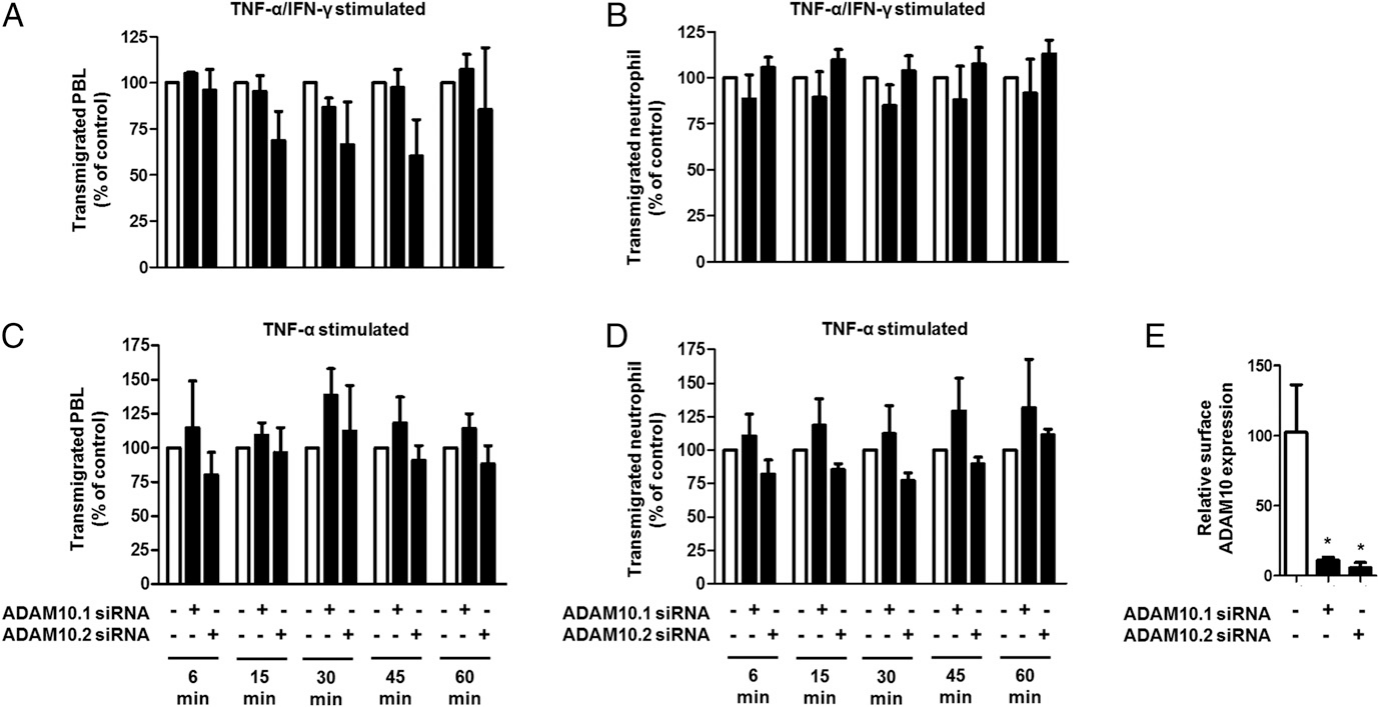
In the presence of neutrophils, knockdown of endothelial ADAM10 does not decrease transmigration of lymphocytes under in vitro flow conditions. HUVECs were transfected with 10 nM negative control (white bars) or one of two ADAM10 siRNA duplexes (black bars). HUVECs were replated into six-well ibidi slides 4 h post transfection, and stimulated with either 100 U/ml TNF-α and 10 ng/ml IFN-γ for 24 h (**A** and **B**) or 100 U/ml TNF-α alone for 4 h (**C** and **D**), prior to perfusing a bolus of freshly isolated human PBLs (fluorescently labeled with CellTracker Orange) and neutrophils (fluorescently labeled with calcein-AM) over the HUVEC monolayers at 0.05 Pa for 4 min. After a 2 min wash with cell-free PBS 0.15% BSA, a single image of one field of view of the HUVEC monolayer was made using time-lapse phase-contrast microscopy at the indicated time points. Cells were classified as firmly adherent or transmigrated. The data were adjusted such that the magnitude of transmigration following ADAM10 knockdown was expressed as a percentage of the negative control knockdown. Of note, the number of PBLs that were recruited on TNF-α–stimulated HUVECs was 60–70% reduced in comparison with the numbers recruited following TNF-α/IFN-γ stimulation. Error bars represent the SEM from three experiments. Data were normalized by arcsine transformation and statistically analyzed by a two-way ANOVA and Bonferroni post hoc comparison test; no significant differences were detected. (**E**) ADAM10 knockdown was confirmed by flow cytometry and quantitated. Error bars represent the SEM from the three experiments in (A)–(D). Data were normalized by arcsine transformation and analyzed by *t* test. *p < 0.05 compared with control siRNA-transfected cells.

To establish a more high-throughput assay of transmigration to facilitate future mechanistic studies, PBL transmigration was assessed under static conditions. Consistent with the studies under flow, ADAM10 knockdown on HUVECs reduced PBL transmigration (by ∼35%), with a consequent increase in the percentage of firmly adherent cells that had not transmigrated (Fig. 2A). To investigate whether a particular major PBL subset was affected by loss of endothelial ADAM10, transmigration of CD19^+^ B lymphocytes and CD4^+^ and CD8^+^ T lymphocytes were assessed, because together they account for ∼80% of PBLs (data not shown). CD4^+^ and CD8^+^ T lymphocytes had reduced transmigration following endothelial ADAM10 knockdown, but CD19^+^ B lymphocytes did not (Fig. 2B). To determine whether reduced PBL transmigration was due to a delay or a complete block, transmigration was allowed to proceed for 60 min, in comparison with the previous time point of 7 min. This confirmed delayed PBL transmigration following endothelial ADAM10 knockdown, because transmigration had reached normal levels after 60 min (Fig. 2C). ADAM10 knockdown efficiency was ∼80% by flow cytometry (Fig. 2D). To determine if impaired PBL transmigration was maintained in the presence of neutrophils, CellTracker Orange-labeled PBLs were mixed with calcein-labeled neutrophils. No defect in PBL transmigration under flow, over a 60-min time course, was observed in the presence of neutrophils, using ADAM10-knockdown HUVECs stimulated with either TNF-α/IFN-γ (Fig. 3A, 3B) or TNF-α alone (Fig. 3C, 3D). ADAM10 knockdown efficiency was 85–90% (Fig. 3E). Taken together, these data show that endothelial ADAM10 can enhance the initial rate of T lymphocyte transmigration under both flow and static conditions, but the requirement for ADAM10 is lost in the presence of neutrophils.

### The transmembrane chemokines CX3CL1 and CXCL16 on HUVECs are not involved in PBL transmigration

**FIGURE 4.**
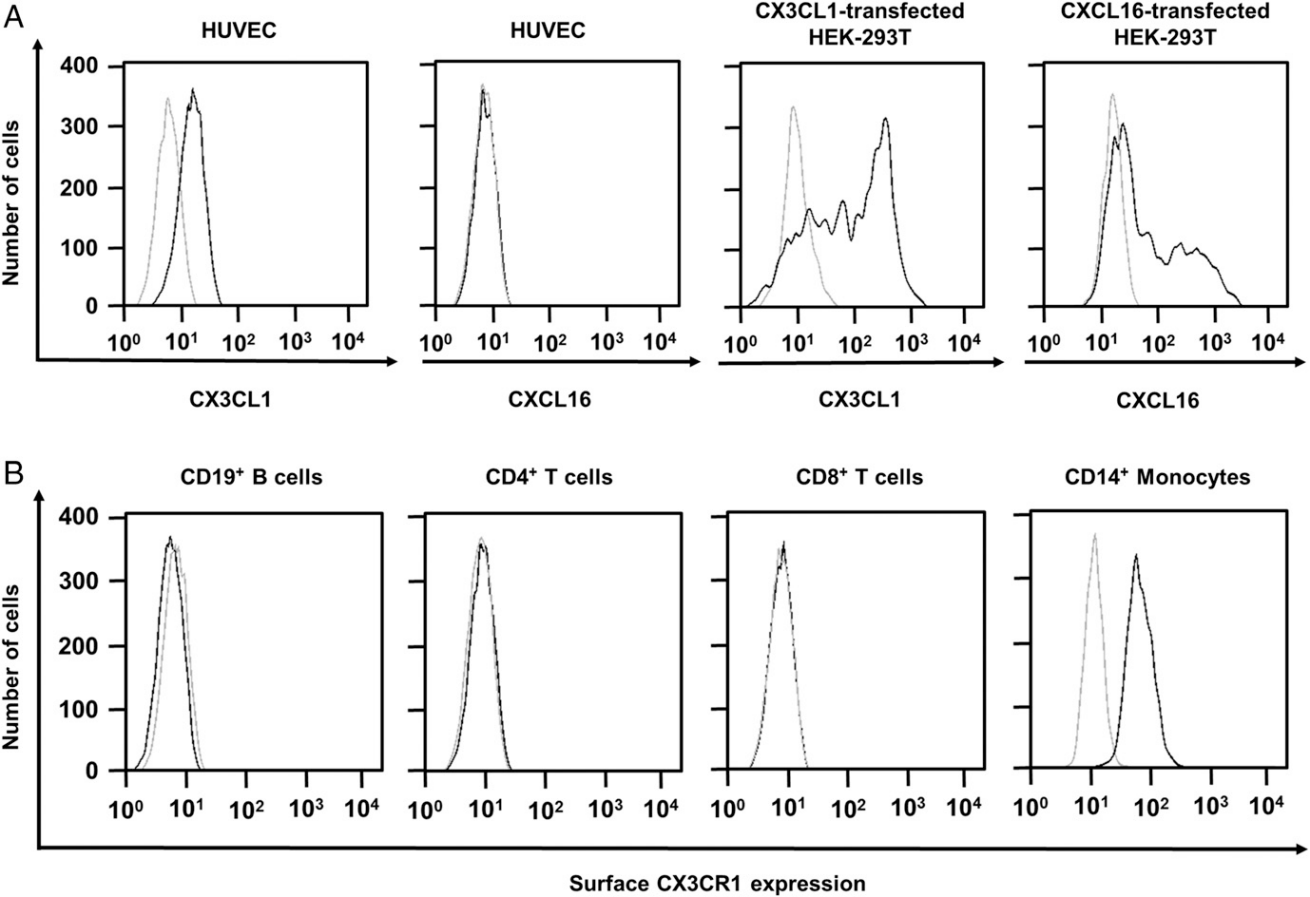
HUVECs express the transmembrane chemokine CX3CL1 but not CXCL16, and lymphocytes do not express the CX3CL1 receptor CX3CR1. (**A**) Expression of CX3CL1 and CXCL16 on HUVECs was assessed by flow cytometry, for which the black line represents chemokine staining and the gray line isotype control staining. HEK-293T cells transiently transfected with CX3CL1 or CXCL16 were used as a positive control, for which the black line represents chemokine-transfected cells and the gray line mock-transfected cells. (**B**) Expression of CX3CR1 on the major PBL subpopulations CD19^+^ B cells, CD4^+^, and CD8^+^ T cells was assessed by flow cytometry. CD14^+^ monocytes were used as a positive control. The black line represents CX3CR1 expression and the gray line isotype negative control staining. All data are representative of three experiments.

The transmembrane chemokines CX3CL1 and CXCL16 are ADAM10 substrates with the potential to regulate PBL transmigration. Indeed, an adhesion assay using primary human leukocytes, adhering to a CX3CL1-transfected epithelial cell line, suggested the possibility that ADAM10 cleavage of CX3CL1 might release captured leukocytes to promote subsequent transmigration (9). However, whether endogenous levels of these chemokines on HUVECs can facilitate the recruitment and subsequent transmigration of PBLs has not previously been demonstrated. To investigate the potential for this, the expression levels of CX3CL1 and CXCL16 were assessed on HUVECs by flow cytometry. HUVECs expressed CX3CL1 but not CXCL16 (Fig. 4A). As a positive control, to confirm the efficacy of the chemokine Abs, both detected their respective targets on HEK-293T cells transiently transfected with human CX3CL1 or CXCL16 (Fig. 4A). To determine if endogenous CX3CL1 on HUVECs had the potential to support the adhesion and transmigration of PBLs, the expression of the CX3CL1 receptor, CX3CR1, was assessed on PBLs by flow cytometry. Gating for the three most abundant PBL subsets, B lymphocytes, CD4^+^, and CD8^+^ T lymphocytes, revealed that CX3CR1 expression was undetectable on these cells (Fig. 4B). As a positive control for the CX3CR1 Ab, CX3CR1 was detected on human monocytes (Fig. 4B). These findings suggest that CX3CL1 and CXCL16 play no role in PBL transmigration across HUVEC monolayers in our model system, because the receptor for CX3CR1 is not expressed on the PBLs and CXCL16 is not expressed on the HUVECs. Thus, regulation of PBL transmigration must be due to another ADAM10-sensitive substrate.

### VE-cadherin expression levels increase in the absence of ADAM10, and partial VE-cadherin knockdown to normal levels rescues the PBL transmigration defect

**FIGURE 5.**
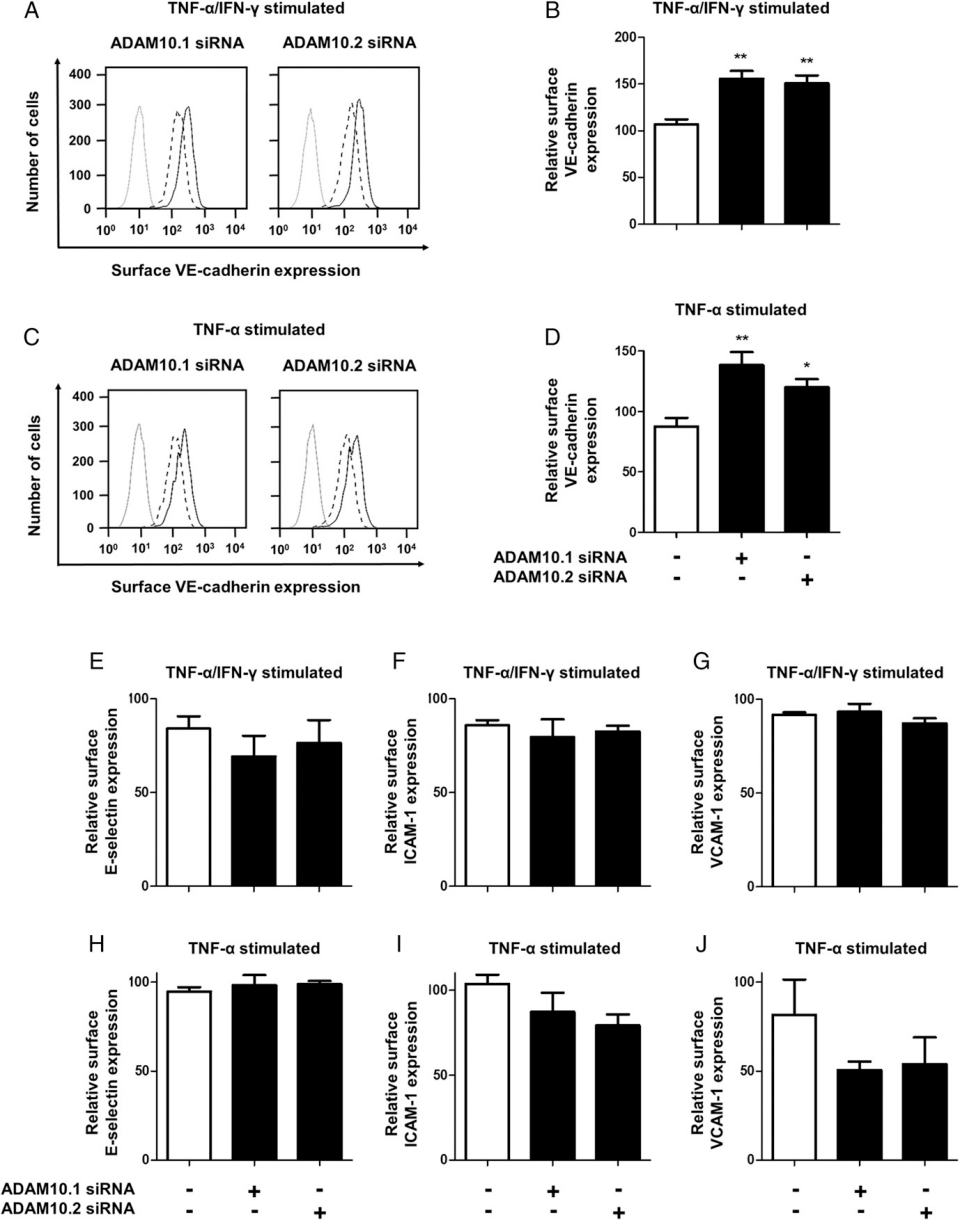
Knockdown of endothelial ADAM10 increases VE-cadherin surface expression but does not affect the surface expression of major cellular adhesion molecules E-selectin, ICAM-1 and VCAM-1. HUVECs were siRNA transfected as described in Fig. 1A and 48 h later stimulated for 24 h with TNF-α/IFN-γ or for 4 h with TNF-α. (**A**–**D**) VE-cadherin expression was measured by flow cytometry. The black line represents VE-cadherin expression on cells following ADAM10 siRNA transfection, the broken line represents VE-cadherin expression on cells following control siRNA transfection, and the gray line is isotype negative control staining. Representative data are shown in (A and C), and quantitated data are shown in (B) and (D). Error bars represent the SEM from four experiments. Data were normalized by arcsine transformation and analyzed by *t* test compared with the control siRNA treatment. *p < 0.05, **p < 0.01. (**E**–**J**) HUVECs were subjected to ADAM10 knockdown as described in (A)–(D), and then surface expression of E-selectin (E and H), ICAM-1 (F and I), and VCAM-1 (G and J) was measured by flow cytometry and quantitated as described in (B) and (D). Error bars represent the SEM from three to four experiments.

**FIGURE 6.**
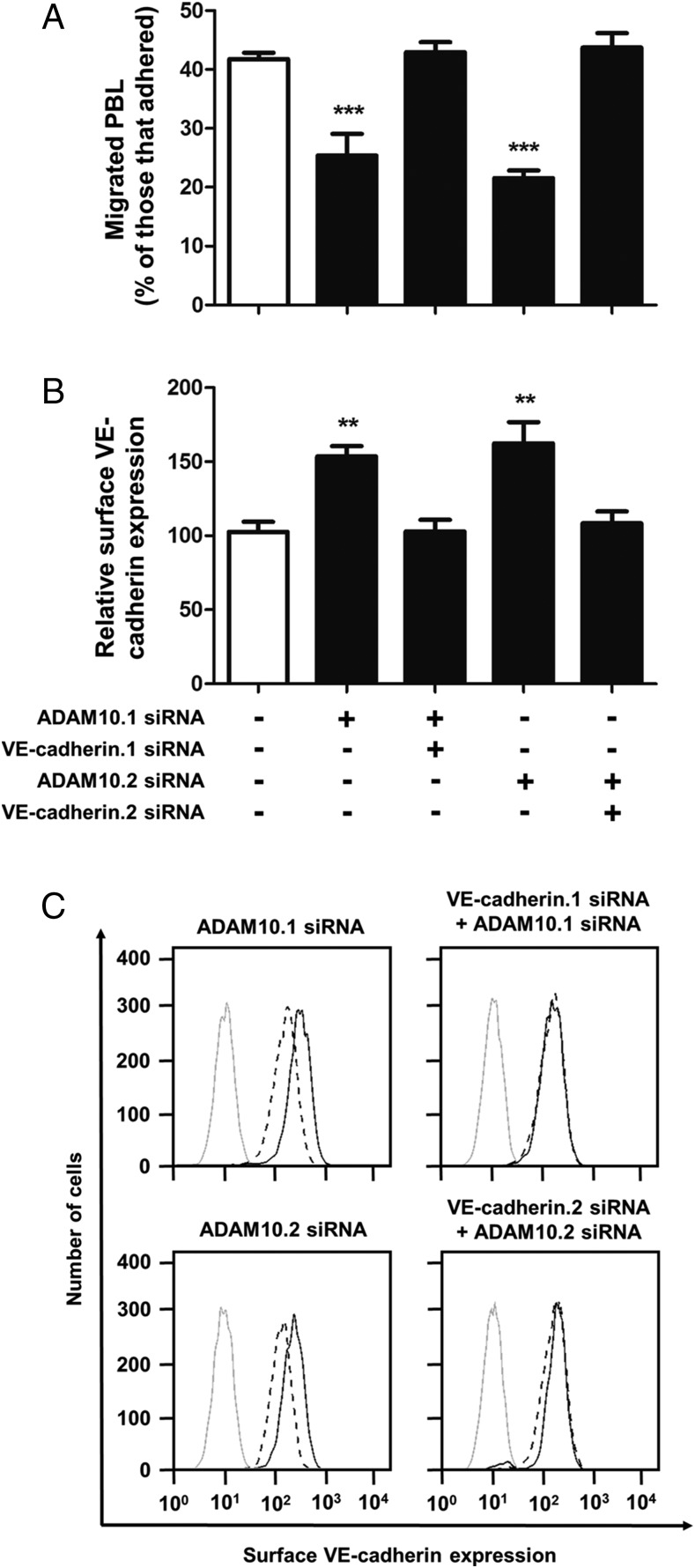
Partial knockdown of VE-cadherin, in combination with full ADAM10 knockdown, restores lymphocyte transmigration. (**A**) HUVECs were transfected with 10 nM negative control siRNA or one of two ADAM10 siRNA duplexes in the presence or absence of one of two VE-cadherin siRNA duplexes at 0.5 nM. PBL static transmigration assays were performed as described in Fig. 2A. Error bars represent the SEM from five independent experiments. Data were normalized by arcsine transformation and statistically analyzed by a two-way ANOVA and Bonferroni post hoc comparisons test. ***p < 0.001 compared with the negative control siRNA transfected data. (**B**) Partial VE-cadherin knockdown was confirmed 48 h post transfection by flow cytometry. Error bars represent the SEM from five independent experiments. Data were normalized by arcsine transformation and statistically analyzed by one-way ANOVA and Dunnett post hoc comparisons test. **p < 0.01 compared with negative control siRNA transfected data. (**C**) Representative flow cytometry histograms from the data quantitated for (B). The black line represents VE-cadherin expression on cells following ADAM10 siRNA transfection, the broken line represents VE-cadherin expression on cells following negative control siRNA transfection, and the gray line is isotype negative control staining. Confirmation of ADAM10 knockdown was assessed by flow cytometry, as explained in the legend to Fig. 1, and found to be ~90% reduced upon quantitation (data not shown).

The ADAM10 substrate VE-cadherin is a homotypic cell adhesion molecule that forms adherens junctions between endothelial cells, and is critical for endothelial barrier function (1–4). Previous studies have shown that ADAM10 inhibition or knockdown decreases shedding of VE-cadherin and decreases endothelial monolayer permeability (5, 6, 28). This is accompanied by a reduction in transmigration efficiency of human T cell blasts (6). However, this study did not assess whether elevated VE-cadherin levels were responsible for the transmigration phenotype. In this study, surface VE-cadherin levels following ADAM10 knockdown on HUVECs were assessed by flow cytometry. Surface VE-cadherin was found to be increased by ∼50% using two different siRNA duplexes, and following treatment with either TNF-α/IFN-γ (Fig. 5A, 5B) or TNF-α alone (Fig. 5C, 5D). In contrast, expression levels of the major endothelial cell adhesion molecules E-selectin, ICAM-1, and VCAM-1 were not significantly affected by ADAM10 knockdown (Fig. 5E–J). To determine whether increased VE-cadherin expression levels were causing the transmigration defect, ADAM10 knockdown was combined with partial VE-cadherin knockdown designed to return VE-cadherin levels to normal. Strikingly, this approach restored PBL transmigration under conditions of ADAM10 knockdown, with one of two different VE-cadherin siRNA duplexes (Fig. 6A). Flow cytometry confirmed that VE-cadherin expression levels had returned to normal following the partial knockdown, and quantitation revealed that this was significant (Fig. 6B, 6C). These results strongly suggest that the elevated VE-cadherin surface expression level is responsible for impaired PBL transmigration in the absence of ADAM10.

### TspanC8 tetraspanins Tspan5 and Tspan17 regulate VE-cadherin expression and promote PBL transmigration

**FIGURE 7.**
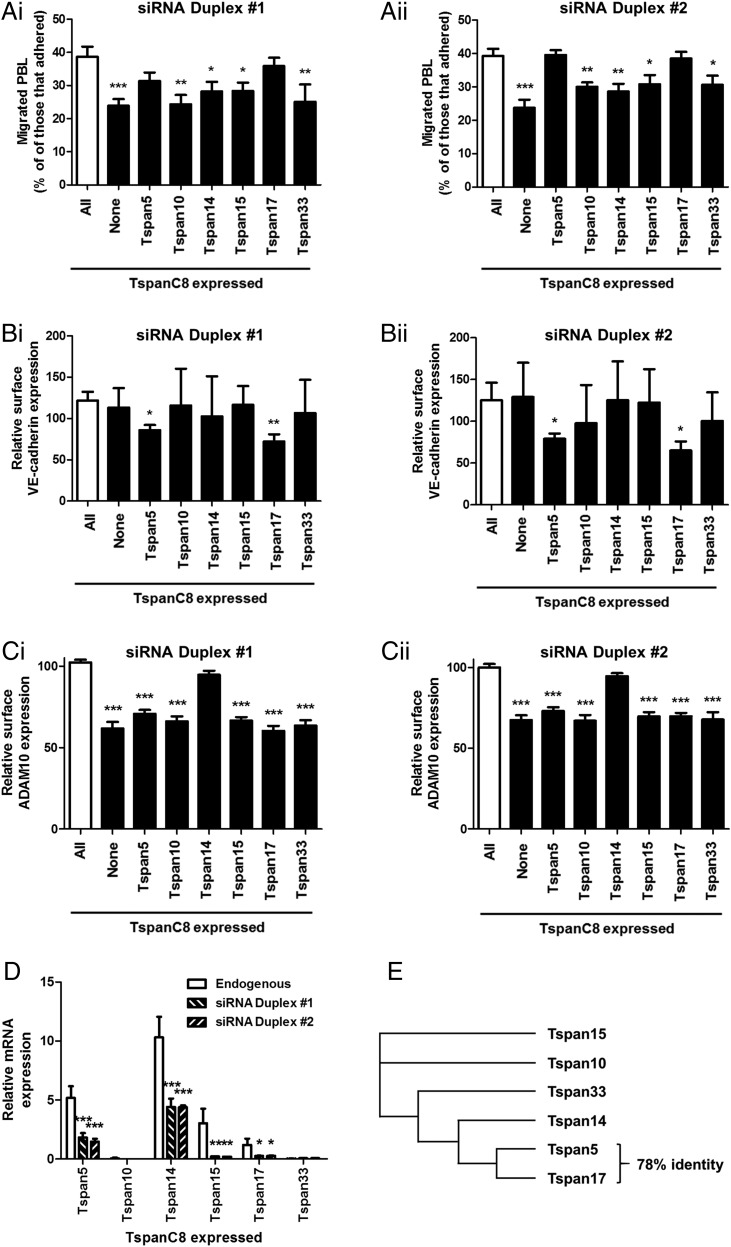
The presence of Tspan5 or Tspan17 on endothelial cells is sufficient to maintain lymphocyte transmigration. (**A**) HUVECs were transfected with negative control siRNA, or 5 nM siRNA to each of the six TspanC8s in combination, or 5 nM siRNA to different combinations of five TspanC8s. Two different siRNA duplexes (i and ii) were used for each TspanC8. (A**i** and A**ii**) PBL transmigration assays were performed as described in Fig. 2A. (**Bi** and B**ii**) VE-cadherin and (**Ci** and C**ii**) ADAM10 surface levels on the transfected HUVECs from (A) were assessed by flow cytometry. Error bars represent the SEM from at least three independent experiments. Data were normalized by arcsine transformation and statistically analyzed by a two-way ANOVA and Bonferroni post hoc comparison test. Data corresponding to siRNA duplex 1 is on the left and data corresponding to siRNA duplex 2 is on the right. *p < 0.05, **p < 0.01, ***p < 0.001 compared with control siRNA transfected cells. (**D**) Knockdown efficiency of TspanC8 tetraspanins was assessed by RT-PCR. Data were statistically analyzed by *t* test. *p < 0.05, **p < 0.01, ***p < 0.001 compared with the negative control siRNA transfected cells. (**E**) Amino acid sequences of human TspanC8s were analyzed by the multiple sequence alignment tool Clustal Ω (37), and the data presented as a dendrogram.

**FIGURE 8.**
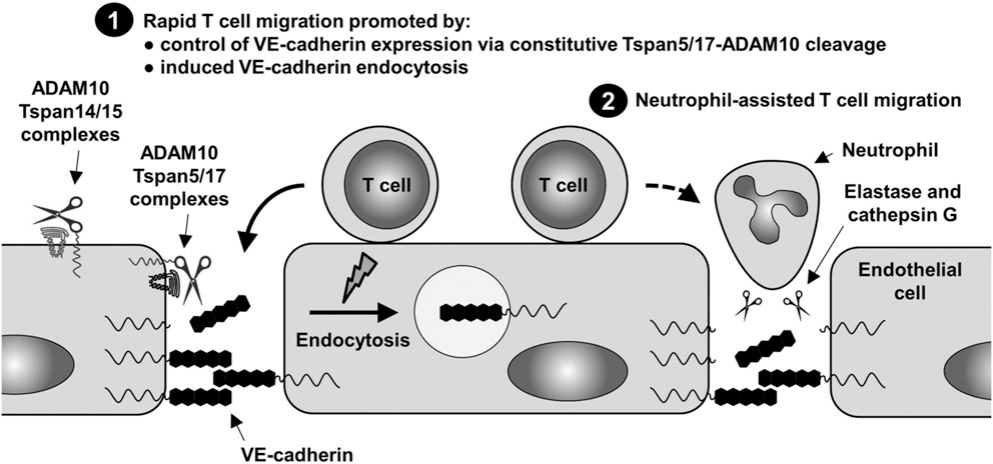
A model figure to show the proposed function of endothelial Tspan5/17-ADAM10 complexes in T cell transmigration. The highly related Tspan5 and Tspan17 promote constitutive ADAM10 shedding of VE-cadherin to control surface expression levels. Other endothelial tetraspanins such as Tspan14 and Tspan15 cannot promote VE-cadherin shedding. (**1**) Upon T cell adhesion to the endothelial monolayer, rapid transmigration is facilitated by VE-cadherin endocytosis. (**2**) Transmigrating neutrophils may overcome the T cell requirement for endothelial ADAM10 via release of elastase and cathepsin G to shed VE-cadherin.

The six TspanC8 tetraspanins have recently emerged as ADAM10-interacting proteins and may traffic the metalloproteinase to distinct subcellular localizations and substrates (21). HUVECs express five TspanC8s, namely Tspan5, 10, 14, 15 and 17; Tspan33 is not detectable (14). To determine whether a specific TspanC8 regulates PBL transmigration, and to overcome potential redundancy between TspanC8s, a combination knockdown approach was undertaken, in which HUVECs were systematically transfected with siRNA duplexes corresponding to all but one of the six TspanC8s. This approach revealed that only the knockdown combinations that retained expression of either Tspan5 or Tspan17, maintained normal PBL transmigration (Fig. 7A). This was accompanied by a reduction in surface VE-cadherin levels (Fig. 7B). This requirement of Tspan5 or Tspan17 to maintain PBL transmigration was not due to increased ADAM10 surface levels (Fig. 7C). Indeed, a partial reduction in ADAM10 was detected when only Tspan5 or Tspan17 were retained, and only Tspan14 expression was sufficient to maintain normal levels of the metalloproteinase (Fig. 7C), which is consistent with the relatively high expression level Tspan14 in HUVECs (14). Knockdown of each tetraspanin was confirmed by RT-PCR (Fig. 7D), because Abs are not available to most TspanC8s. Knockdown was not complete, but varied from ∼55% for Tspan14 to 90% for Tspan15 (Fig. 7D). Interestingly, CLUSTAL Ω protein sequence analysis showed that Tspan5 and Tspan17 are the two most highly related TspanC8s, sharing 78% identity in humans (Fig. 7E), providing an explanation for their common role revealed in this study. Collectively these results suggest that Tspan5 and Tspan17, which have not been studied functionally before, are novel facilitators of T lymphocyte transmigration through their regulation of ADAM10 and VE-cadherin (Fig. 8).

## Discussion

This study reports three main findings that provide insights into the molecular mechanism of leukocyte transmigration. First, endothelial ADAM10 promotes transmigration of T lymphocytes, but not B lymphocytes or neutrophils, in an in vitro flow assay. Second, this function of ADAM10 is mediated via its substrate VE-cadherin, but not CX3CL1 and CXCL16. Third, Tspan5 and Tspan17 regulate VE-cadherin expression and promote lymphocyte transmigration, but the other four ADAM10-interacting TspanC8 tetraspanins do not.

The previously proposed role for endothelial ADAM10 in leukocyte transmigration is based on in vitro transwell assays in the absence of flow (6, 10, 11). One of these studies showed a positive role for endothelial ADAM10 using unstimulated HUVECs at passage two to four, following ADAM10 knockdown or inhibition, and cultured human T cell blasts (6). A second study reported a positive role for endothelial ADAM10 using HUVECs up to passage four, stimulated with IFN-γ and TNF-α and subjected to ADAM10 knockdown, and a CX3CR1-transfected mouse B cell line (11). However, in both of these studies, the cells used may transmigrate by mechanisms that are different to those employed by blood-borne cells, which are the physiologically relevant populations to study in the context of leukocyte trafficking during inflammation. A third study showed that endothelial ADAM10 facilitates transmigration of freshly isolated human neutrophils in response to CXCL8, using lung human microvascular endothelial cells subjected to ADAM10 knockdown and used at passage five to six (10). Such assays that use artificially generated chemotactic gradients across unstimulated endothelial cell monolayers are difficult to interpret. Appropriate adhesion molecules may be absent and the endogenous and sequential signals that regulate the trafficking of neutrophils after recruitment from flow are unlikely to be operative. To highlight the problems inherent in interpreting such assays, we have previously shown that CXCL8 plays no role in the migration of human neutrophils across TNF-α–stimulated HUVECs (29). Thus, to our knowledge the current study is the first to demonstrate a requirement for endothelial ADAM10 under flow conditions using freshly isolated primary lymphocytes from peripheral blood. The HUVECs were at a relatively low passage one and were stimulated with IFN-γ and TNF-α to mimic inflammatory conditions. The requirement for endothelial ADAM10 was observed for T lymphocytes but not B lymphocytes or neutrophils, and it will now be important to determine whether this holds true in vivo. For such an experiment, the endothelial-specific ADAM10 knockout mouse (30) will be required, because of the embryonic lethality of the whole-body ADAM10 knockout.

Schulz et al. (6) identified VE-cadherin as an ADAM10 substrate and demonstrated that transmigration of T cell blasts was dependent on endothelial ADAM10. VE-cadherin is a homotypic cell-cell adhesion molecule and a major component of adherens junctions, and functions as a critical regulator of endothelial permeability and leukocyte transmigration (1–4). Together, these data prompted Schulz et al. (6) to suggest that VE-cadherin shedding was necessary for T cell transmigration, although they acknowledged that their data did not prove this. In the current study, ADAM10 knockdown resulted in an almost 50% increase in cell surface VE-cadherin expression and almost 50% impaired lymphocyte transmigration, consistent with the hypothesis of Schulz et al. (6). This was due to a delay in transmigration, because the phenotype was observed at a 7-min time point but was no longer evident at 60 min. To determine whether elevated surface VE-cadherin was responsible for impaired transmigration, ADAM10 knockdown was combined with partial VE-cadherin knockdown, to return VE-cadherin levels to normal. Remarkably this restored lymphocyte transmigration, strongly suggesting that elevated surface VE-cadherin was responsible for impaired transmigration. The two most likely other ADAM10 substrates that could have been regulating transmigration were the transmembrane chemokines CX3CL1 and CXCL16 (7–9), but these were ruled out by the lack of detectable CXCL16 on HUVECs, and the lack of the CX3CL1 receptor (CX3CR1) on lymphocytes. Our CX3CR1 data are consistent with a previous report of minimal expression on freshly isolated lymphocytes, although expression can be strikingly induced after 5 d of in vitro polarization in IL-2 (31). Taken together these data suggest that, in this in vitro model system, impaired lymphocyte transmigration in the absence of endothelial ADAM10 is a consequence of elevated surface VE-cadherin, which would provide a stronger barrier to lymphocyte transmigration. The fact that transmigration in the absence of ADAM10 can be restored to normal, by reducing VE-cadherin to normal levels, suggests that ADAM10 activity is not required to shed VE-cadherin during transmigration. Indeed, in the currently proposed model of transmigration (1–4), the leukocyte provides an outside-in signal to loosen adherens junctions by removing VE-cadherin molecules from the path of the leukocyte. The precise mechanism by which this occurs is not clear and may differ depending on the vascular bed and activation stimuli. Dephosphorylation of a key tyrosine residue in the VE-cadherin cytoplasmic tail is reported to promote VE-cadherin endocytosis in a mouse model (32). In another study, endocytosis is not observed in cultured primary human endothelial cells; instead tyrosine phosphorylation of the VE-cadherin tail is associated with displacement of VE-cadherin in an undefined way (33). Also important is the interaction between VE-cadherin and catenin proteins, which link the adhesion molecule to the actin cytoskeleton. Overexpression of p120-catenin in cultured human endothelial cells increases VE-cadherin expression and stability at the cell surface, reducing leukocyte transmigration (33). Similarly, in a mouse model, a VE-cadherin fusion construct with α-catenin promotes the stability of endothelial cell junctions and impairs leukocyte transmigration (34). To incorporate our findings with these data, we propose that in the absence of ADAM10, the elevated VE-cadherin levels lead to a delay in VE-cadherin endocytosis and/or displacement, with the result that T lymphocyte transmigration is delayed.

A requirement for neutrophil ADAM10 in neutrophil transmigration has been demonstrated in vitro and in vivo, albeit without a definitive mechanism (35). The role for endothelial ADAM10 in neutrophil transmigration is less clear, given that the current study suggests that it is not required, but that of Dreymueller et al. (10) suggests that it is. A possible explanation is the different assays employed to measure transmigration. The major differences in the Dreymueller study were the endothelial cells used, specifically their origin, passage number and lack of cytokine stimulation, and the use of transwells with CXCL8 as a chemoattractant (10). Therefore, endothelial ADAM10 may be required for neutrophil transmigration under some circumstances but not others. A potential mechanism to explain a less important role for endothelial ADAM10 in neutrophil transmigration, versus lymphocyte transmigration, is the specific expression of neutrophil elastase and cathepsin G, two proteases that can cleave VE-cadherin during neutrophil transmigration (36). These may thus overcome the requirement for endothelial ADAM10. Interestingly, when neutrophils were mixed with lymphocytes, no impaired transmigration of the latter was observed across ADAM10-deficient endothelium. This suggests that lymphocyte transmigration can be assisted by neutrophils, possibly mediated via neutrophil elastase and cathepsin G cleavage of VE-cadherin.

Recent studies have shown that ADAM10 interacts with six different tetraspanin transmembrane proteins, Tspan5, 10, 14, 15, 17, and 33, which are related by protein sequence and termed the TspanC8s (13–15, 18, 20). Individual tetraspanins function by interacting with specific partner proteins and regulating their intracellular trafficking and lateral mobility and clustering at the cell surface (16, 17). Indeed, TspanC8s are essential for ADAM10 exit from the ER (13–15) and different TspanC8s have distinct subcellular localizations (13, 18), promote ADAM10 shedding of distinct substrates (13, 18, 20), and may cause ADAM10 to adopt distinct conformations (20). Therefore, ADAM10 can be regarded as six different molecular scissors, depending on the associated TspanC8 (21). HUVECs have previously been shown to express mRNA for Tspan5, 10, 14, 15, and 17, but not Tspan33 (14); effective Abs are not available to most TspanC8s. To determine which TspanC8(s) might be important for the role of endothelial ADAM10 in lymphocyte transmigration, a systematic knockdown of the six different combinations of five TspanC8s was performed in HUVECs. Despite TspanC8 knockdown efficiencies of only 55–90% in these experiments, this approach showed that expression of either Tspan5 or Tspan17 was sufficient to maintain lymphocyte transmigration. In contrast, all knockdown combinations that knocked down both Tspan5 and Tspan17 yielded impaired lymphocyte transmigration. This Tspan5/17 effect was not due to any specific impact on ADAM10 surface expression, but did correlate with VE-cadherin surface levels, consistent with the earlier finding that VE-cadherin provides a mechanistic explanation for the role of endothelial ADAM10 in lymphocyte transmigration. Tspan5 and Tspan17 are largely unstudied TspanC8s; the only studies to date have shown Tspan5 to promote ADAM10-mediated Notch activation and osteoclast formation in vitro (13, 18, 19). However, protein sequence alignments indicate that they are the two most closely related tetraspanins, with the human forms sharing 78% amino acid identity. This suggests redundant roles for Tspan5 and Tspan17 in regulating ADAM10 and VE-cadherin levels. Future studies will aim to determine whether Tspan5 and Tspan17 localize ADAM10 into close proximity to VE-cadherin, and/or promote an ADAM10 conformation favorable to VE-cadherin shedding.

In summary, and taking together all the available literature on the role of TspanC8s, ADAM10, and VE cadherin in leukocyte trafficking, we now propose the following model to explain how T cell transmigration is regulated by these molecules (Fig. 8). Endothelial Tspan5/ADAM10 and Tspan17/ADAM10 complexes constitutively shed VE-cadherin to maintain an optimal expression level. When T cells bind to endothelial cells to initiate transmigration, VE-cadherin is endocytosed to allow the T cell to migrate through endothelial junctions. Although such endocytosis does not require ADAM10 activity, excessive basal VE-cadherin expression levels in the absence of Tspan5/ADAM10 and Tspan17/ADAM10 complexes will delay the process. Finally, our data using combinations of leukocytes strongly implies an alternative route of T cell migration that is assisted by neutrophils, whereby proteases on migrating neutrophils render the endothelial cell pool of ADAM10 redundant by rapidly cleaving VE-cadherin to allow efficient T cell transmigration.
